# Effects of Teucrium polium Ethyl acetate Extract on Serum, Liver and Muscle Triglyceride Content of Sucrose-Induced Insulin Resistance in Rat

**Published:** 2012

**Authors:** Seyyedeh Elaheh Mousavi, Ali Shahriari, Akram Ahangarpour, Hossein Vatanpour, Abbas Jolodar

**Affiliations:** a *Department of Biochemistry, Veterinary faculty of Shahid Chamran University, Ahwaz. Iran.*; b *Department of Biochemistry, Veterinary faculty of shahid Chamran University, Ahwaz. Iran.*; c *Department of Physiology, Diabetes and Physiology Research Center, School of Medicine, Jundishapour Medical Sciences University, Ahwaz, Iran.*; d *Department of Molecular Genetic, Veterinary Faculty of Shahid Chamran University, Ahwaz. Iran.*; e *Department of Toxicology and Pharmacology, Pharmacy School Medicine, University of Shahid Beheshti, Tehran, Iran.*

**Keywords:** *Teucrium polium*, Insulin resistance, Triglyceride, Sucrose, Rat

## Abstract

Possessing putative hypolipidemic effects, *Teucrium polium (TP*) have been traditionally used as a medicinal plant in Iran. The aim of the present study was to investigate this effect on the sucrose-induced insulin resistance male rat model.

Thirty Wistar male rats weighting 180 **± **20 g were divided into five groups of six each. Four groups were given sucrose 50% in drinking water for 10 weeks. In 8^th^ week of treatment, three groups of them were randomly selected and treated with *Teucrium polium *(*T. polium) *ethyl acetate extract (50, 100 and 200 mg/Kg for two weeks). Control animals were fed using normal rat chow. After ten weeks, blood samples were collected from the heart. Blood Glucose, insulin, leptin, lipid content and fasting insulin resistance index (FIRI) as well as liver and muscle glycogen and lipid contents were determined. Final data were analyzed by ANOVA and post-hoc Tukey’s test.

Liver glycogen contents and blood levels of glucose and insulin were significantly increased in high sucrose (HS) group compared with control group. A significant decrease was observed in blood glucose and insulin levels, FIRI, serum total lipid, triglyceride and VLDL-c as well as the liver triglyceride level, muscle and liver glycogen contents in 100 and 200 mg/Kg of TP-treated groups compared with HS group. Leptin level was significantly decreased in 50 and 100 mg/Kg groups compared with HS group.

The treatment with *T. polium *ethyl acetate extract (TP-EAE) induced a dose-dependent reduction in serum, liver and muscle triglyceride (TG) and liver glycogen content levels, as well as serum insulin. These effects may be attributed, in part, to the hypolipidemic effect of *TP *flavonoids; otherwise, the hepatoprotective and antioxidant activity of TP-EAE may improve the liver function and reverse harmful sucrose effects.

## Introduction

An individual with insulin resistance who has relative rather than absolute insulin deficiency afflicted type 2 diabetes. Insulin deficiency leads to failure of glucose consumption in diabetes mellitus (DM) and consequently results in breakdown of lipids and proteins. DM induces a group of syndromes characterized by insulin resistance and hyperglycemia – altered metabolism of lipids, carbohydrates and proteins – and an increased risk of cardiovascular disease complications (1). High carbohydrate diets and excessive total calories are associated with much higher Triglyceride content in serum, liver and muscle (2). Some evidence suggests that an excess accumulation of hepatic and skeletal muscle lipid is associated with insulin resistance and type 2 diabetes mellitus in human (3-6) and animal models (7, 8). Dietary sugar or sucrose content is the major focus of carbohydrate-based dietary guidelines. Sucrose consumption, especially its fructose component produces pre-diabetic status. After the absorption in the gastrointestinal tract, fructose is transported via the portal circulation to the liver, where it enters hepatocytes via the glucose transporter GLUT5-independently of insulin (9, 10). Phosphofructokinase, a hepatic enzyme that governs glycolysis in liver, negatively regulates glucose breakdown while fructose can evade this rate-limiting control mechanism and is metabolized into glycerol-3-phosphate and acetyl-coenzyme A. These two intermediate metabolites are then used as substrates for glyceride synthesis, contributing to very low-density lipoprotein triglyceride production in the liver (11, 12). The exposure of liver to such large quantities of fructose leads to rapid stimulation of lipogenesis and triglyceride accumulation, which in-turn contributes to reduced insulin sensitivity and hepatic insulin resistance/glucose intolerance (11).

Many herbal formulations have been recommended for the treatment of diabetes as an alternative for the currently available therapeutic options like oral hypoglycemic agents and insulin therapy (13). In the field of alternative medicine, *Teucrium Polium L. *(*Labiatea*) is known to have hypoglycemic effects and it is widely suggested to the diabetic patients in Iran and throughout the world. *T. polium *(Calpoureh) is a member of Labiatea family which are well-known to possess antibacterial, anti-inflammatory (14-16), antidiabetic and hypoglycemic (17-20), antihypertensive (1) and antilipidemic (21) activities. Unfortunately, most aqueous and organic extracts of the plant are hepatotoxic (22). There are few reports relating to the hepatoprotective and antioxidant effect of *T. polium *ethyl acetate extract (23, 24). Both properties are very useful in controlling diabetes. To our knowledge, there are a few reports about the hypotriglyceridemic effect of TP-EAE on carbohydrate-induced hypertriglyceridemia, so this study was conducted to investigate the effect of TP-EAE on serum, muscle and liver lipid profiles, also insulin resistance in pre-diabetic rats.

## Experimental

*Plant material*

*Teucrium polium *L. (Lamiaceae) samples were collected from Izeh local area, Khuzestan, Iran. The dried leaves of *Teucrium polium *were authenticated by Faculty of Agriculture, Shahid Chamran University of Ahwaz, Iran.

*Preparation of plant extract*

Fresh leaves of *Teucrium polium *were separated, cleaned and dried at room temperature. The dried powdered plant material (300 g) was extracted by continuous mixing with ethanol (70% and 80%), at room temperature for 24 h. After filtration, ethanol was evaporated until only water remained. Water phase was subsequently extracted with ethyl acetate, then filtered and concentrated under the vacuum condition up to a concentration of 1 g /1 mL of extract (23, 25).

*Experimental animals*

Healthy adult male Wistar rats weighting 180 20 ± g were purchased from Physiology Institute of Ahvaz (Iran). Animals were housed in cages under the conditions of controlled temperature (25°C), relative humidity of 65 ± 10% and a 12 h artificial light period for 10 weeks and had free access to water and standard pellet diet. The experiments were carried out after the approval of protocol by Ahwaz Institutional Animals and according to the current guidelines of the laboratory animals’ care.

*Experimental design*

After 7 days of acclimatization, animals were divided randomly into five equal groups, four of which were given sucrose 50% in drinking water for 10 weeks. The control group consisted of 6 animals which received enough water and food and were left on treated. At the end of 8^th^ week, high sucrose groups were treated with oral administration of ethyl acetate extract of *T. polium *at doses of 50, 100 and 200 mg/Kg for two consecutive weeks (26).

*Biochemical analysis*

24 h after the last administration, animals were euthanized and the blood samples were collected from the heart and centrifuged at 3500 rpm for about 20 min, then serum was separated out and blood glucose, leptin, insulin and fasting insulin resistance index (FIRI), glycogen and lipid profile in serum as well as in liver and skeletal muscle were determined. Serum High-density lipoprotein cholesterol (HDL-c), total cholesterol (TC) and triglyceride (TG) were determined using commercial kits and enzymatic assays.

Serum total lipid (TL) was measured by chemochromatography and sulfo-phospho-vanillin reaction. The extracted liver and muscle TG contents were estimated through Neri-Frying method (27).

*Very low-density lipoprotein cholesterol (VLDL-c) estimation*

Very low-density lipoprotein cholesterol (VLDL-c) equals one-fifth of TG content. To calculate the amount of low-density lipoprotein cholesterol (LDL-c), the sum of HDL-c and VLDL-c cholesterol content is subtracted from total cholesterol amount and the remnant is LDL-c.

*Hormonal assays*

Blood samples were collected daily between 08:30 and 10:30 am under fasting conditions. All concentrations were determined in duplicate through ELISA (Labor Diagnostika Nord GmbH, Germany) for leptin and IRMA (BioSource Europe S.A) for insulin. Intra- and inter-assay coefficients of variation were 4.3 and 5.8% for leptin and also 6 and 6.1% for insulin, respectively. Low-end sensitivities for leptin and insulin were 0.5 ng/mL and 1 μIU/mL, respectively.

*Blood glucose level and fasting insulin resistance index (FIRI)*

Serum Glucose level was measured by kinetic (enzymatic) and colorimetric methods using Glucose Estimation Kit (Pars Azmoon, Iran). Fasting insulin resistance index (FIRI) was calculated according to the formula (28).

$\mathrm{FIRI}=\frac{\mathrm{fasting insulin}(\mathrm{mU}/\mathrm{mL})\times \mathrm{fasting glucose}(\mathrm{mg}/\mathrm{dL})}{25}$

*Liver and muscle glycogen content*

Liver and skeletal muscle Glycogen content was estimated using Russell and Taylor’s method. In this process, potassium ions help breaking down the glycogen to glucose subunits, which in-turn produce a colored complex with phenol-sulfuric acid that is present in the environment. The intensity of the produced color is proportional to the amount of glycogen in the specimen (29).

*Extracting liver and muscle lipids using Hara and Radin method*

The muscle and liver tissue slices weighting 1 g were obtained and grinded using laboratory pounder. Samples were then transferred to the tubes containing 9 mL of isopropanol : hexane (3 : 2) mixture and 5 glass ball bearings. The mixture was then kept in room temperature for 8 h and homogenized using a shaker machine. The lipid content of the cells was extracted and solved in the organic phase. The samples were centrifuged at 3000 rpm for 10 min and cellular debris was sedimented. The solved cellular extract remained in organic phase. Cellular extract was transferred to the other tubes using pasture pipette. A volume of 12 mL sodium sulphate solution was added to each tube in order to remove non-lipid materials. The samples were kept in room temperature for 1 min. Sodium sulphate solution was prepared with adding the dried salt to 150 mL of distilled water. After adding the solution, two distinct phases appeared in each tube; the upper phase (hexane-rich layer) transferred to the other tube using pasture pipette. Using an air pump, the organic solvent was evaporated and the lipid content remained in the tube and finally the extract was air-dried. A volume of 500 μL isopropanol was added to each tube to re-solve the dried lipids and was shaken for 5 min. The remnant material was used to measure the TG, total cholesterol and lipids (30).

*Statistical analysis*

The results are presented as the mean ± SD. Data was analyzed through the SPSS program. Comparison between the groups was done using ANOVA and their significance was established via post-hoc test (Tukey). Differences in the range of p < 0.05 were considered statistically significant.

## Results and Discussion

Our results indicated that serum levels of glucose, insulin as well as liver glycogen content, TG and VLDL-c were significantly increased in high source group (HS group) compared to control group (p < 0.05). A significant decline was observed in blood glucose and FIRI (p < 0.05) in TP-treated groups compared to HS group. The observed effect, at 100 and 200 mg/Kg, was more obvious than the 50 mg/Kg groups (Table 1). On the other hand, FIRI was increased in HS group compared to the TP-treated groups. However, leptin levels tend to increase in HS group but did not changed significantly in comparison with the control group. TP-EAE administration at doses of 50 and 100 mg/Kg for two weeks, caused significant (p < 0.05) reduction in leptin level (Table 1). Three doses of extract caused significant reduction in the insulin level and liver glycogen content compared to the HS group (Tables 1 and 2). Muscle glycogen content was significantly decreased in 100 and 200 mg/Kg TP-treated groups in comparison with other groups (Table 2). 

**Table 1:** Effect of different doses of *Teucrium polium *ethyl acetate extract on serum glucose, insulin, leptin and lipid parameters in rats fed by sucrose-rich diet. (In all group n=6, values are mean ± SD;*= p < 0.05).

**parameter**	**Control **	**HS **	**HS+50mg/kg**	**HS+100mg/kg**	**HS+200mg/kg**
**Glucose (mg/dL)**	14.102±17.261 ^b*^	189.983±24.248 ^ade*^	159.175±23.628	148.738±3.808 ^b*^	121.818±17.078 ^b*^
**Insulin (μIU/mLl)**	3.120±0.486 ^b*^	6.888±2.190 ^acde*^	3.073±0.464 ^b*^	2.972±0.443 ^b*^	3.130±0.270 ^b*^
**Leptin (ng/dL)**	1.133±0.053	1.453±0.073 ^cd*^	1.087±0.131 ^b*^	1.260±0.247 ^b*^	1.083±0.247
**FIRI**	26.705±3.995	34.328±10.445 ^de*^	23.800±4.400	18.652±1.758 ^b*^	14.310±2.209 ^b*^
**Total Cholesterol (mg/dL)**	59.705±13.350^bcde* ^	80.490±15.321^acde*^	85.000±10.849^ab^*	82.158±8.777^ab*^	86.667±18.040^ab*^
**Total Lipid (mg/dL)**	608.000±35.355	747.000±143.979^de*^	773.000±109.545^de*^	541.750±75.870^bc*^	526.750±109.349^bc*^
**Triglyceride (mg/dL)**	64.000±9.977^b*^	100.257±17.498^ade*^	92.713±11.490^de*^	67.897±4.732^bc*^	74.920±19.167^bc*^
**HDL-cholesterol (mg/dL)**	42.130±7.323	36.180±7.346 ^c^*	53.188±10.089^b*^	48.697±4.690	45.840±9.749
**LDL-cholesterol (mg/dL)**	46.809±7.147	40.243±6.186 ^d*^	34.650±6.795 ^de*^	60.816±11.378^bc^*	52.604±8.910 ^c*^
**VLDL-cholesterol (mg/dL)**	12.800±1.996^bc*^	20.51±3.500^ade*^	18.543±2.298^ade*^	13.410±0.952^bc*^	13.606±2.636^bc*^

N = 6; values are mean ± SD; *p < 0.05

**Table 2:** Effect of different doses of *Teucrium polium *ethyl acetate extract on liver and skeletal muscle glycogen content in rats fed by sucrose-rich diet. (In all group n = 6, values are mean ± SD;*= p < 0.05).

Glycogen (mg/g of wet tissue)			
**Liver**		**Muscle**	
Control	33.546±6.704 ^b*^		4.113±0.415^de*^
HS	41.517±3.857 ^acde*^		4.587±0.140^de*^
HS+50mg/kg	32.448±5.513 ^b*^		3.727±0.474^de*^
HS+100mg/kg	18.902±2.556 ^b*^		2.545±0.593^ab*^
HS+200mg/kg	24.087±1.928 ^b*^		2.810±0.337^ab*^

a = difference between control group with the other groups. b = difference between high sucrose-induced group with the other groups. c = difference between dose 50 mg/kg extract+sucrose-induced group with the other groups. d=difference between dose 100 mg/kg extract + sucrose-induced group with the other groups. e = difference between dose 200 mg/kg extract+sucrose-induced group with the other groups.

N = 6; mean ±SD, *p < 0.05.

**Figure 1:**
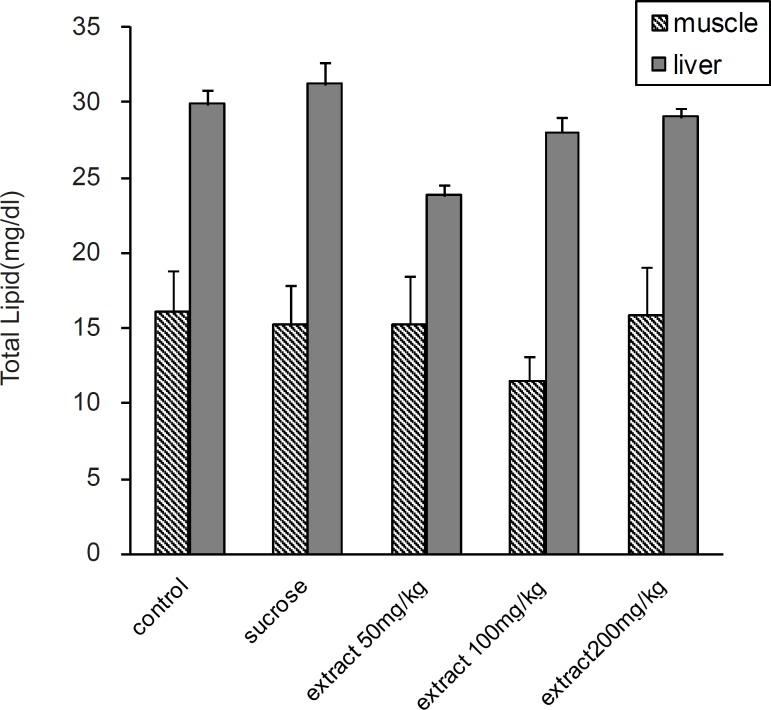
Effect of different doses of *Teucrium polium *ethyl acetate extract on liver and skeletal muscle lipid content in rats fed by sucrose-rich diet. Each value represents the mean ± SD (n = 6). All values statistically different *p < 0.05

**Figure 2:**
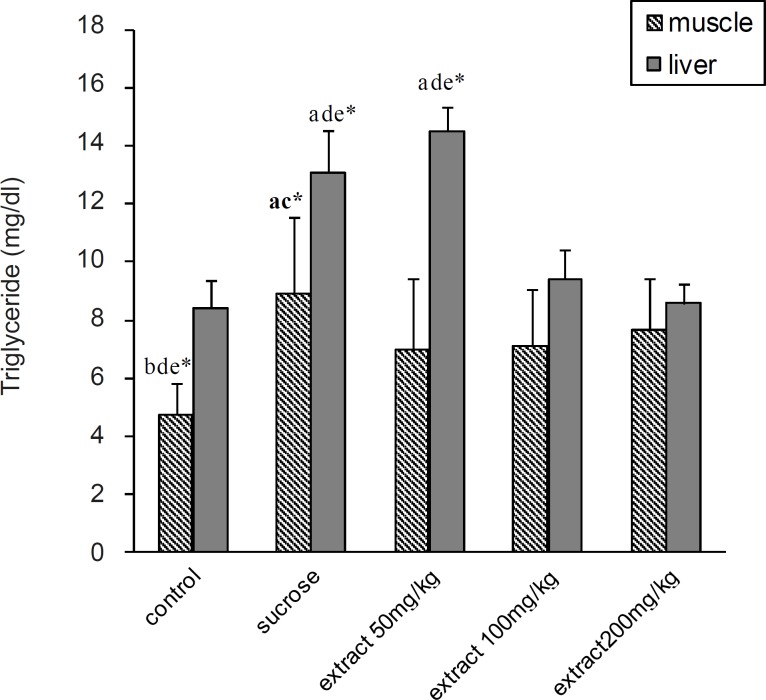
Effect of different doses of *Teucrium polium *ethyl acetate extract on liver and skeletal muscle triglyceride content in rats fed by sucrose-rich diet. Each value represents the mean ± SD (n = 6). All values statistically different *p < 0.05

Serum total cholesterol was significantly increased in all experimental groups compared to the control group (p < 0.05). In 100 and 200 mg/Kg groups, there was a significantly decrease in serum TG, VLDL-C and TL compared to 50 mg/Kg and HS groups (p < 0.05). According to the results, TG was significantly increased in HS and 50 mg/Kg groups compared to the control group (p < 0.05). At 100 and 200 mg/Kg doses, there was also significantly decreases in liver TG (p < 0.05) compared to the HS group (Figure 2). But there was no significant difference in liver and muscle TL between the groups (Figure 1). 

In addition, the muscle TG content was significantly increased in HS, 100 and 200 mg/Kg groups in comparison with the control group (p < 0.05). TP-EAE at a dose of 50 mg caused a significant decrease in muscle TG compared to the HS group (Figure 2). 

However, HDL-c level tended to be increased in the TP-treated groups compared to the HS group, the effect of which was significant just at dose of 50 mg/Kg (Table 1). LDL-c level was significantly increased in 100 mg/Kg group compared to the HS group (p < 0.05), however; there was a significant decrease in 50 mg/Kg group compared to the 100 and 200 mg/Kg groups (Table 1). 

Treating hyperglycemic rats with *T.polium *extract induced a significant (p < 0.05) dose-dependent reduction in the serum glucose level in comparison with the control animals; but the difference was significant at 100 and 200 mg/Kg groups. Furthermore, a dose-dependent reduction in the serum insulin level as well as liver glycogen content was observed in TP-treated groups. The results show a dose-dependent reduction in the serum level of leptin in TP-treated groups in comparison with HS group, but the difference was significant at 50 and 100 mg/Kg groups. Hypolipidemic activities of *T. polium *ethyl acetate extract induced a significant (p < 0.05) dose-dependent reduction in the serum and liver lipid content compared to the HS group, but the difference was more significant at 100 and 200 mg/Kg doses.

The increasing of serum glucose and insulin as well as FIRI, is associated with the pre-diabetic and insulin resistance status in the HS group. Numerous studies showed that a high fructose (HF) and/or HS diet induces the insulin resistance in rodents (31-32). The pathogenesis of insulin resistance which is caused using a of insulin resistance which is caused using a HF and/or HS is unclear. It was reported that the excessive circulating free fatty acids and glucose may contribute to the insulin resistance (33 ,34) .

in addition , in HS diets , the exposure of the liver to large quantities of fructose activate Phosphofructokinase, a hepatic enzyme that governs glycolysis in liver ,negatively regulates the glucose can evade this rate-limiting control mechanism and is metabolized into glycerol-3-phosphate and acetyl-coenzyme A. These two intermediate metabolites that are used as substrates for glyceride synthesis, contribute to the VLDL-c triglyceride production and accumulation in the liver, which in-turn contribute to the reduce insulin sensitivity and hepatic insulin resistance/glucose intolerance (11, 12).

High-carbohydrate diets may raise triglyceride levels by different mechanisms, such as hepatic overproduction of VLDL-c triglycerides and its secretion into the circulation (35, 36), reduction of lipoprotein lipase activity (37, 38) or retardation lipolysis of triglyceride-rich lipoproteins (39).Furthermore, VLDL-c is the precursor to LDL-c and its overproduction may lead to cardiovascular complications (40, 41). A similar effect was observed in our study; serum VLDL-c level was increased in HS group. On the other hand, TP-EAE was able to produce a dose-dependent reduction in VLDL-c level at 100 and 200 mg/Kg doses. An increase in serum VLDLL-c content elevates the LDL-c level; therefore, carbohydrate-rich diets increase the serum level of LDL-c (42, 43), but in our study, there was no change in serum LDL-c level. Our results showed that the serum LDL cholesterol level is increased in TP-treated groups (100 and 200 mg/Kg) that is consistent with Shahraki *et al. *(2007) reports who demonstrated that serum cholesterol, triglyceride and LDL-c levels were increased through *T. polium *aqueous extract in Streptozotocin-induced diabetic male rats (44).

Moreover, a primary finding indicated that the higher level of liver and muscle TG content in HS group was directly associated with the insulin resistance. In this regard, many evidences suggest that an excess accumulation of hepatic (3, 4) and skeletal muscle lipid is associated with insulin resistance in human obesity and in type 2 diabetes mellitus (5, 6) and animal models (7, 8).Hypotriglyceridemic effect of TP-EAE at the present study is in agreement with Rasaekh *et al. *(2001) findings on rat (21). For the first time, we have shown that hypotriglyceridemic effect of TP-EAE at doses of 100 and 200 mg/ Kg is associated with the reduction in the liver and TG muscle content as well as declination in serum insulin and glucose. Although the lipid-lowering mechanism remain to be understood, *T. polium *contains a wide range of active pharmacologic agents including alkaloids, glycosides, terpenoids, sterols, triterpenes, and flavonoids (45, 46). TP-EAE is a flavonoid-rich extract. Flavonoids may have insulin like and/or insulin-triggering properties have been extracted from the plant. Some kinds of flavonoids may omit the lipid synthesis and secretion from liver (47). Reduction of TG in liver concurrent with hypotriglyceridemia in TP-treated groups may indicate preventive effect of these flavonoids on liver-TG synthesis and its secretion to blood circulation. Reinner *et al. *(1989) investigated the effects of *T. polium *fractions on blood cholesterol and TG levels in diabetic male rats and found that the plant has fat-lowering effects by decreasing the blood cholesterol level. Cholesterol lowering effect is largely due to the inhibition of its absorption in small intestine and promoting its hepatic release. The liver plays a critical role in discharging the cholesterol via bile secretion. (48).

According to Kadifkova *et al. *(2007) and Ardestani *et al. *(2007) results, ethyl acetate extracts used in this study have hepatoprotective and antioxidant effects (23, 24) that may protect the liver from the harmful effect of fructose and improve the function of liver.

High sucrose diets increased the serum levels of glucose, insulin as well as liver glycogen content in our work. Glucose transportation and subsequent activation of glycogen synthase are the important steps for controlling the rate of glycogen accumulation in insulin-sensitive tissues such as skeletal muscle (30, 49). Therefore, observed increase in liver glycogen synthesis in HS group may be explained by the availability and high levels of insulin, even in the face of insulin resistance. Treatment with *T. polium *ethyl acetate extract (100 and 200 mg/Kg) for 14 days decreased the muscle and liver glycogen content indicating that the defective glycogen storage of the diabetic rats was partially corrected by the extract. Therefore, it may be concluded that *T. polium *increases the glycogenolysis rate by decreasing the hepatic glycogen content or postponing the absorption of blood glucose as a result of blood insulin level decline.

Yazdanparast *et al. *(2005) showed that *Teucrium polium *extract may reduce the high blood glucose levels through enhancing insulin secretion by the pancreas without significant metabolic changes in Streptozotocin-induced diabetic male rats (20).

In our study, the amount of leptin was decreased in TP-treated groups. Leptin secretion via adipocytes is stimulated by insulin and the plasma leptin significantly correlates with insulin serum concentrations (50). Thus, the decreasing effect of *T. polium *on plasma insulin level may play a role in leptin reduction. 

Leptin has important actions in stimulating the vascular inflammation, oxidative stress, and insulin resistance which may contribute to the pathogenesis of type 2 diabetes mellitus, atherosclerosis, and the coronary heart disease (51, 52). So, possessing the lowering effects on leptin, *T. polium *may improve these conditions.

## Conclusion

The *Teucrium polium *ethyl acetate extract modulates the serum, liver and muscle TG, and improves the insulin resistance in the experimental rat fed by sucrose-rich diet which may be useful in preventing or early treatment of diabetic disorders. However, further studies are needed to determine possible mechanisms of action, but these effects may be attributed in part to hypolipidemic effect of *T. polium *flavonoids, otherwise, the hepatoprotective and antioxidant activity of TP-EAE may improve the function of liver and reverse the harmful sucrose effects.
